# Data reduction for spectral clustering to analyze high throughput flow cytometry data

**DOI:** 10.1186/1471-2105-11-403

**Published:** 2010-07-28

**Authors:** Habil Zare, Parisa Shooshtari, Arvind Gupta, Ryan R Brinkman

**Affiliations:** 1 Department of Computing Science, University of British Columbia, Vancouver, BC, Canada; 2 Terry Fox Laboratory, BC Cancer Agency, 675 W 10th Ave., Vancouver, BC, Canada; 3 Faculty of Science, University of British Columbia, Vancouver, BC, Canada; 4 Department of Medical Genetics, University of British Columbia, Vancouver, BC, Canada

## Abstract

**Background:**

Recent biological discoveries have shown that clustering large datasets is essential for better understanding biology in many areas. Spectral clustering in particular has proven to be a powerful tool amenable for many applications. However, it cannot be directly applied to large datasets due to time and memory limitations. To address this issue, we have modified spectral clustering by adding an information preserving sampling procedure and applying a post-processing stage. We call this entire algorithm SamSPECTRAL.

**Results:**

We tested our algorithm on flow cytometry data as an example of large, multidimensional data containing potentially hundreds of thousands of data points (*i.e*., "events" in flow cytometry, typically corresponding to cells). Compared to two state of the art model-based flow cytometry clustering methods, SamSPECTRAL demonstrates significant advantages in proper identification of populations with non-elliptical shapes, low density populations close to dense ones, minor subpopulations of a major population and rare populations.

**Conclusions:**

This work is the first successful attempt to apply spectral methodology on flow cytometry data. An implementation of our algorithm as an R package is freely available through BioConductor.

## Background

High throughput data analysis is a crucial step in research endeavours involving gene expression, protein classification, and flow cytometry. A classical approach for analysing biological data is to first group individual data points based on some similarity criterion, a process known as clustering, and then compare the outcome of clustering with the biological hypotheses. An example of this approach is in the analysis of flow cytometry data where populations of cells that express specific intracellular or surface proteins are identified. Flow cytometry is a technique for measuring physical, chemical and biological characteristics of individual microscopic particles such as cells and chromosomes. It has many applications in molecular and cell biology for both clinical diagnosis and research purposes [1]. In cytometers, cells are individually passed through a laser beam and the scattered light is captured to measure up to 19 characteristic of each cell [2]. As thousands of cells can be analyzed per second, cytometers can generate large-sized datasets. Recently, sophisticated methods have been developed for automatic analysis of flow cytometry data [3-5]. The proposed clustering techniques include: mixture modeling approach [6], model-based cluster analysis [7], feature-guided clustering [8], density-based clustering [9], combining the curvature information with density information [10], and image processing [11]. The automatic techniques are useful in clinical and research applications such as: application of high-content flow cytometric screening (FC-HCS) to the problem of cellular signature definition for acute graft-versus-host-disease [12], vaccine trials [13], visualizing data in stem cell research [14], and immunophenotypic characterization of B-cell chronic lymphoproliferative disorders (B-CLPD) [15].

### Problem Statement

Automated identification of flow cytometry cell populations is complicated by overlapping and adjacent populations, especially when low and high density populations are close to each other. Analysing such data requires clustering methods that can separate these populations. Non-parametric methods include density clustering [16], real-time adaptive clustering [17], and Kohonen self-organizing maps [18]. The application of these methods is restricted since the first two are subjective due to a dependency on user-defined thresholds, and the latter one requires the number of clusters to be determined by the user. While accurately determining the number of clusters may not be a key issue in some clinical cytometry analysis [19], this requirement can be a critical obstacle for other analyses such as identifying novel populations for biomarker discovery [3].

Model-based clustering techniques such as FLAME [20], flowClust [21] and flowMerge [22] have been developed to improve results. flowMerge uses the flowClust framework to identify clusters based on a t-mixture model methodology, followed by a merging step to account for overestimation of the number of clusters by the Bayesian information criterion. FLAME uses a skew t-mixture model, which is in theory more robust to skew, because unlike t-distributions, skew t-distributions can be asymmetric [20]. However, the running time of this algorithm increases with the fourth degree of the number of dimensions. In practice this tends to make the algorithm impractical for more than five dimensions, while flow cytometry data can have up to 19 dimensions. Overall, the major drawback of these parametric methods is the requirement for assumptions on either the size of the clusters or the cluster distributions and shapes [23], which could result in incorrect identification of biologically interesting populations. In addition, one challenge for existing approaches is the identification of rare populations. Spectral clustering is a non-parametric clustering method that avoids the problems of estimating probability distribution functions by using a heuristic based on graphs [24]. It has proved useful in many pattern recognition areas [25-28]. Not only does it not require *a priori *assumptions on the size, shape or distribution of clusters, but it has features that make it particularly well-suited to clustering biological data:

• It is not sensitive to outliers, noise or shape of clusters;

• It is adjustable so that biological knowledge can be utilized to adapt it for a specific problem or dataset;

• There is mathematical evidence to guarantee its proper performance [29].

Two main challenges in applying spectral clustering algorithm on large data sets are the computationally expensive steps of constructing the normalized matrix and computing its eigenspace. For instance, for high throughput biological data containing one million data points (*i.e*., vertices), it requires computing eigenspace of a million by million matrix, which is infeasible in terms of memory and time. Although there are some approximation methods for speeding up this computation [30,31], these could produce undesired errors in the final results. The problem of applying this algorithm on large datasets has been studied in [32] using Nyström's method. They suggest a strategy of sampling data uniformly, clustering the sampled points and extrapolating this solution to the full set of points. However, sampling data uniformly can miss low-density populations entirely when the density of adjacent populations varies considerably, a situation that often arises for biologically interesting populations in flow cytometry data. Appendix 3 includes an experiment to explain the effect of uniform sampling in such cases.

Data reduction schemes have been developed to reduce the complexity of the flow cytometry data while preserving the information [33,34]. These methods reduce the dimensionality but not the size of the dataset, the latter being the more important bottleneck for spectral clustering.

### Our Approach

We hypothesized that spectral clustering could significantly improve high throughput biological data analysis. However, serious empirical barriers are encountered when applying this method to large data sets. Specifically, for *n *data points, the running time is *O*(*n*^3^), requiring *O*(*n^2^*) units of memory. For instance, it would take 2 years and 5 terabytes of memory to analyze a typical flow cytometry sample with 300,000 events. We developed a novel solution for this problem through our non-uniform information preserving sampling. Our heuristic approach is specific to cytometry applications and made it possible for the first time, to apply spectral clustering method on flow cytometry data.

## Results

In this paper, we distinguish between the terms biological populations, clusters and components as follows. A *population *is a set of cells with similar functionality or molecular content. By a *cluster*, we mean a set of data points that are grouped together by spectral clustering algorithm. We incorporate a post-processing stage on spectral clusters to find the *connected components *intended to estimate the biological populations.

### Algorithm

#### Spectral Clustering

The first step is to build a graph. The vertices represent the *n *data points (*e.g*., cells in flow cytometry data), and the edges between the vertices are weighted based on some *similarity *criterion. The adjacency matrix of the graph is then normalized using the following formula:

(1) $$\overset{ƒ}{A}=D^{-\frac{1}{2}}AD^{-\frac{1}{2}},$$

where *A *is the adjacency matrix of the graph and *D *is a diagonal matrix where the (*i*, *i*) entry is equal to the sum of the weights on the edges that are adjacent to vertex *i*.

The next step is to compute eigenspace of the normalized matrix. That is, all vectors *V_i _*and values *λ_i _*satisfying the following equation are computed:

(2) $$\overset{ƒ}{A}{\vec{V}}_{i}=\lambda_{i}\vec{V}.$$

In order to find *k *clusters, an *n *by *k *matrix is built using the *k *eigenvectors with highest eigenvalues. The rows of this matrix are normalized and finally k-means is used to cluster the rows.

However, the above method cannot be directly applied to flow cytometry data due to large number of data points (cells) per sample. Our solution for this problem is a data reduction scheme developed specifically for this purpose. This reduces the number of vertices significantly, but in a way such that biological information can be preserved by updating the weights on the edges.

#### Data Reduction Scheme

**Figure 1:**
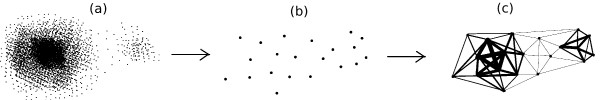
**Data reduction scheme**. (a) Running spectral clustering is impractical on data that contains thousands of points. (b) Faithful sampling picks up a reasonable subset of points such that running spectral clustering is possible on them. However, all information about the local density is lost by considering only these sample points. (c) We assign weights to the edges of the graph; the edges between the nodes in denser regions are weighted considerably higher. The information about the local density is retrieved in this way.

**Figure 2:**
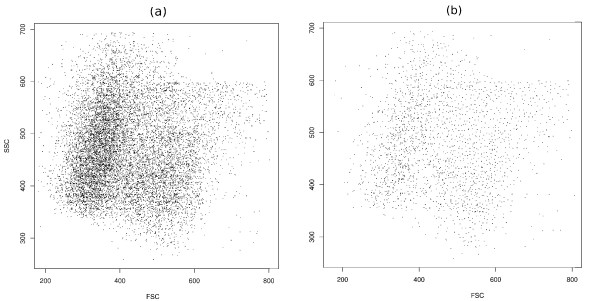
**Faithful sampling**. (a) Original data from telomere data set before sampling. (b) The distribution of representatives is almost uniform in the space after faithful sampling.

While data size can be reduced by known sampling methods [35], a very delicate method should be used to preserve biologically important information. From a high-level perspective, our data reduction scheme (Figure 1) consists of two major steps; first we sample the data in a representative manner to reduce the number of vertices of the graph (Figure 1b). Sample points cover the whole data space uniformly (Figures 2b), a property that aids in the identification of both low density and rare populations. In the second step as described below, we define a similarity matrix that assigns weights to the edges between the sampled data points. Higher weights are assigned to the edges between nodes in dense regions so that information about the density is preserved (Figure 1c).

Faithful Sampling Algorithm

1. Label all data points as *unregistered*.

2. **repeat**

3.    Pick a random unregistered point *p * {the representative of a new community}

4.    Label all unregistered data points within distance *h *from *p *as *registering*

5.    Put registering points in a set called *community p*

6.    Relabel registering points as *registered*

7. **until **All points are registered

8. **return **All communities

After faithful sampling is completed, the set of all representatives can be regarded as a sample from the data. Reducing the value of parameter *h *will increase the number of sample points, resulting in increased computation time and required memory. Conversely, increasing *h *will result in fewer sample points that may lead to too low a resolution. In such a case, the computed spectral clusters may fail to estimate the real cell populations appropriately. In our implementation, we use an iterative procedure (explained in the overview of our algorithm) to adjust *h *automatically such that the number of representatives will be in range 1500-3000. As a result of this adjustment, the following two objectives are achieved. First, computing the eigenspace of a graph with a number of points in this range is feasible, (it takes less than one minute by a 2.7 GHz processor.) Second, the communities are "small" (Figure 2) and the resulting resolution is high enough such that no biologically interesting information is lost.

In the sampling stage, there is no preference in picking up the next data point, therefore, the final distribution of the sampled points will be uniform in the "effective" space. That is, the representatives are distributed almost uniformly in the space where data points were present (Figure 2). As a consequence, by repeating sampling procedure the final results of clustering will not change significantly. This observation is confirmed quantitatively in Appendix 1. By considering just the representatives, density information is effectively ignored so working directly with these representatives results in improper outcome. On the other hand, some biological information from the original data is preserved by the above algorithm that can be retrieved to guide the clustering algorithm. More precisely, for each sample point, we know the list of all points in its neighbourhood (*i.e*., the *members *of the corresponding community). In the next stage, we use this information to define the similarity between two sample points to modify the behaviour of spectral clustering. In this sense, our sampling scheme is *faithful*, meaning that the valuable biological information from the original data points is preserved even after sampling. We call the overall procedure, which consists of faithful sampling, computing modified similarity matrix and spectral clustering, SamSPECTRAL clustering.

#### Similarity Matrix

In this study, we use the following heat kernel formula [36] to compute the similarity between two vertices *i *and *j:*

(3) $$s_{i,j}=e^{-\frac{D^{2}(p_{i},p_{j})}{2\sigma^{2}}},$$

where D (*p_i_, p_j_*) is the Euclidean distance between them. *σ *is a scaling parameter that controls how rapidly similarity between *p_i _*and *p_j _*falls off with increasing distance. We define the similarity between two communities *c *and *c' *as the sum of all pairwise similarities between all members of the first community and all members of the second community. That is,

(4) $$S_{c,c^{\prime }}=\sum_{i\in c}\sum_{j\in c^{\prime }}s_{i,j},$$

where *i *and *j *are members of *c *and *c' *respectively.

We do not normalize the similarity by dividing the above sum by the size of communities because we would lose valuable biological information that is supposed to be preserved. In short, the size of the communities determines the local density of the data points, which is biologically of great importance.

**Figure 3:**
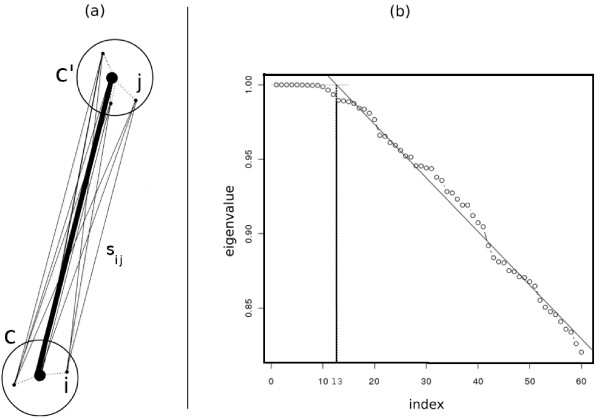
**Defining the similarity between two communities and identifying the number of clusters**. (a) We define the similarity between two communities *c *and *c' *as the sum of pairwise similarities between the members of *c *and the members of *c'*. (b) This figure shows the largest eigenvalues of a sample from the stem cell dataset. The number of clusters is estimated according to the knee point of eigenvalues curve. This point is defined as the intersection of the above regression line and the line *y *= 1. The horizontal coordinate of the knee point estimates the number of spectral clusters.

The above definition is motivated by the following intuition from potential theory that explains how biological information is preserved after faithful sampling by assigning similarities in this way. The eigenvectors of a graph are interpreted as potential functions on the electric network modeled by the graph [37]. Assuming the radius of each community is small enough, the potential values of the community members are almost the same. On the other hand, in potential theory, the equivalent conductance between a group of nodes {*v_i_*} with equal potential values and another group of nodes {*w_j_*} that also have equal potential values is computed by the summation of pairwise conductance between nodes *v_i _*and *w_j _*for all *i *and *j*. Since in our model, the similarity between two vertices is equivalent to the conductance between the corresponding electrical nodes, it is reasonable to sum up pairwise similarities to estimate the equivalent similarity between communities (Figure 3a).

#### Number of Clusters

The number of clusters must be determined before running the spectral clustering algorithm [38]. To find this number automatically and in an efficient manner, we propose a method that is motivated by the following observation from spectral graph theory:

**Theorem **[39]: The number of connected partitions of a graph is equal to the number of eigenvectors with eigenvalue 1.

We observed that typically for flow cytometry data, if *σ *is adjusted properly as explained in the SamSPECTRAL package vignette [40], the first few eigenvalues are close to one and at a point we call *knee point *they start to decrease almost linearly. We compute the knee point by applying linear regression to the eigenvalues curve (Figure 3b) and use the horizontal coordinate of this point as a rough estimate for the number of spectral clusters.

#### Combining Clusters

Applying spectral clustering on sampled data results in graph partitioning, which is almost optimum in the sense of having minimum normalized cut [41,42]. However, in some cases, a biologically interesting population might be split into two or more smaller clusters by SamSPECTRAL. We addressed this issue by adding a post-processing stage wherein the partitions of a population are combined based on known properties of flow cytometry cell populations. Typically, biologically meaningful cell populations in flow cytometry data have their highest density at the centre, and their density decreases towards the border of the population. Since higher density regions indicate communities with relatively more members, the conductance between them is expected to be relatively higher (Equation 4). Thus, similarity between communities is higher in regions with higher densities and the highest similarity is expected to be at the centre of the biological population. This observation forms the basis for our criterion for combining clusters. Specifically, similarity between communities determines the weight on graph edges and we define the maximum weight of the edges of a spectral cluster as *within similarity *of that cluster. Also, the maximum weight of the edges between two different spectral clusters is defined as *between similarity*. If the ratio of *between similarity *to *within similarity *is greater than a predefined threshold (*separation factor*), we conclude that these clusters are partitions of a single population, and should combine them to form a component. We repeat this stage until no two components can be combined. The final components computed in this way are called connected components of the data, and estimate the real biological populations. With smaller separation factors, spectral clusters tend to combine more often.

#### Overview of SamSPECTRAL Algorithm

In summary, the stages of our algorithm are as follows, assuming the data contains *n *points in a *d *dimensional space of volume *V*, and the parameters *m *(max number of communities), *σ *(scaling parameter), and separation factor are set properly.

1. Sampling:

(a) Let $h=\frac{1}{2}\sqrt[{d}]{\frac{V}{m}}$.

(b) **Repeat:**

• Run faithful (biological information preserving) sampling algorithm. Suppose *m' *communities are built.

• Update: $h=h\left(\sqrt[{d}]{\frac{m^{\prime }}{m}}\right)$.

**Until **$\frac{m}{2}\leq m^{\prime }\leq m$.

2. Compute the similarities between communities by adding pairwise similarities *s_i_*, *_j _*defined by **3:**

(5) $$S_{c,c^{\prime }}=\sum_{i\in c}\sum_{j\in c^{\prime }}s_{i,j}.$$

3. Build a graph wherein each community is a vertex. Put edges between all pairs of vertices and weight them by similarity between corresponding communities.

4. Analyze the spectrum of the above graph to find the clusters;

(a) Normalize the adjacency matrix of the graph according to Equation 1.

(b) Compute the eigenspace of the graph and set *k*, number of clusters, according to the knee point of eigenvalues curve.

(c) Run classical spectral clustering algorithm to find *k *clusters.

5. Combine the clusters to find connected components:

(a) Initiate the list of components equal to the list of spectral clusters.

(b) **Repeat:**

• For any pairs of components *C_i_*, *C_j _*, set:

(6) $$\text{separation}\_\text{ratio}:=\frac{\text{between}\_\text{simi}1\text{arity}(C_{i},C_{j})}{{\text{within}}_{\_}\text{simi}1\text{arity}(C_{i},C_{j})}$$

• For each component *C_i_*, compute:

$$M(i):=\underset{j\neq i}{\max}(\text{separation}\_\text{ratio}(C_{i},\;C_{j}))$$

• If for all *i*, *M*(*i*) ≤ separation_factor, **break**.

• Pick an *i *such that *M*(*i*) > separation_factor and let:

$$j=\underset{j\neq i}{\text{arg}\max}(\text{separation}\_\text{ratio}(C_{i},C_{j}))$$

• Combine *C_i _*and *C_j _*, then update list of components.

**Until **number of components > 1.

In the sampling stage, we start with the initial value $h=\frac{1}{2}\sqrt[{d}]{\frac{V}{m}}$ for the neighbourhood. *m *is a parameter that controls *m'*, the final number of sample points such that $\frac{m}{2}\leq m^{\prime }\leq m$. Since in our implementation, we use Manhattan metric to measure the distance between points, the volume of a community can be estimated by $\sigma_{xx}^{1}=0.08,\;\sigma_{yy}^{1}=0.30,\;\sigma_{xy}^{1}=\sigma_{yx}^{1}=0$. Therefore if the the data points were distributed uniformly in the space, we would get *m *sample points in the first run. However, in practice, we need to repeat the procedure after updating the neighbourhood value. According to our experiments, a few iterations are enough to fulfil the terminating condition $\frac{m}{2}\leq m^{\prime }\leq m$. As the running time of this part of SamSPECTRAL is *O*(*nm*), which is negligible compared to eigenspace computation time, we did not attempt to optimize the sampling loop.

#### Modified Markov Clustering Algorithm (MCL)

Step 4 in the above algorithm is the classic spectral clustering method. This step potentially could be substituted by any clustering algorithm for weighted graphs. To verify that our approach is extensible in this sense, we substituted classic spectral clustering with Markov Clustering (MCL) [43] keeping the rest of our algorithm, sampling and post-processing steps, unchanged.

MCL finds the partitions of a graph by simulating flow on the nodes. Simulation is done by iteratively multiplying two type of matrices that correspond to *expansion *and *inflation *operations [43]. Because flow and eigenspace of a graph are strongly related^1^, the outcome of this approach tends to be similar to spectral clustering through computing eigenspace.

### Testing

**Figure 4:**
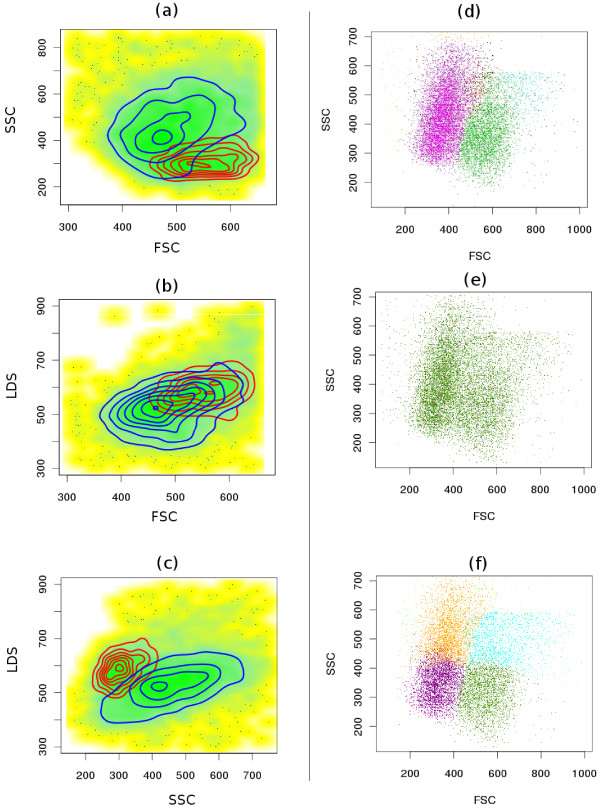
**Comparative clustering of the telomere dataset**. (a-c) Proper identification of overlapping populations. Although two populations shown by red and blue contours are overlapping in all bi-variant plots of this 3-dimensional sample, SamSPECTRAL can properly distinguish them by considering multiple parameters simultaneously.(d) SamSPECTRAL can also identify two major subpopulations of granulocytes correctly, as verified by expert analysis. (e) flowMerge does not distinguish between two populations of interest, and (f) FLAME improperly splits the same sample into several clusters.

**Figure 5:**
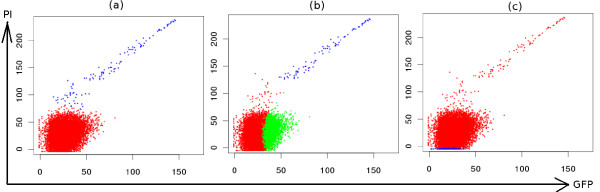
**Comparative clustering of dead cells (PI positive) and live cells (PI negative) in the viability data**. (a) SamSPECTRAL could distinguish between dead cells (blue) and live cells (red) properly. (b) flowMerge identified dead cells correctly, but split live cells into two clusters. (c) FLAME did not distinguish between these two population.

**Figure 6:**
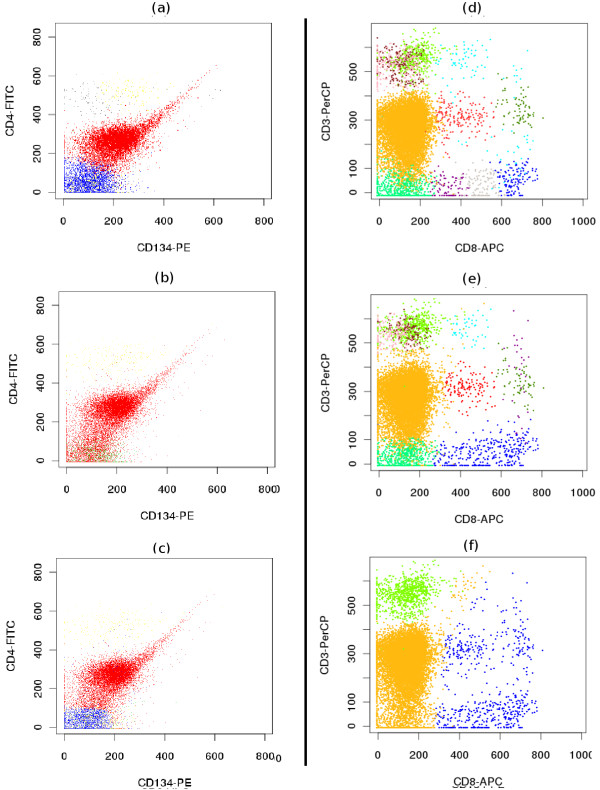
**Comparative clustering of the GvHD dataset**. (Left) Identification of non-elliptical shaped populations. (a) SamSPECTRAL could properly identify the red, non-elliptical population, while (b) flowMerge mixed this population with the one below it. (c) FLAME produced satisfactory results in identifying this population. (Right) Identification of low density populations close to dense populations. (d) SamSPECTRAL and (e) flowMerge could identify the low density population shown in red at the centre of the figure correctly, while (f) FLAME merged this population with the other ones surrounding it.

**Figure 7:**
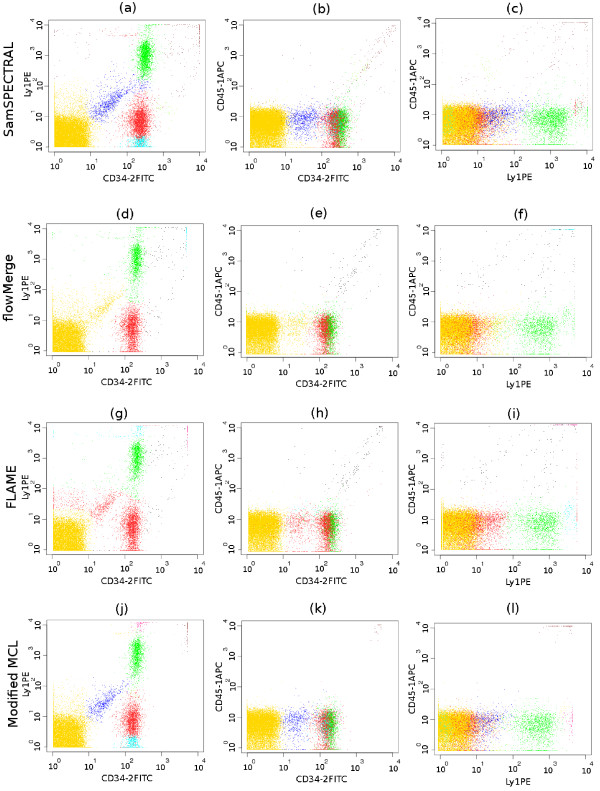
**Comparative identification of a low density population surrounded by much denser populations in the stem cell data set**. (a-c) SamSPECTRAL correctly identified the blue, low density population, while (d-f) flowMerge merged it to the yellow, high density population. (g-i) FLAME merged it to the red population. (j-l) The outcome of our modified MCL was similar to that obtained by SamSPECTRAL using classic spectral clustering. This shows that SamSPECTRAL is extensible by substituting classic spectral clustering with other clustering algorithms for weighted graph.

**Figure 8:**
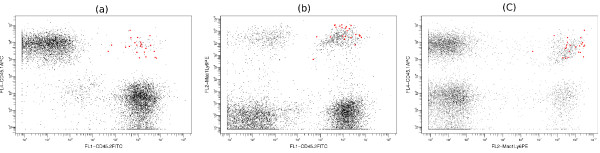
**Rare population in the stem cell data set**. (a-c) This is a typical sample from the stem cell data set that contains a rare population. In these three dimensional plots, the red dots represent the cells that are positive for all three markers. Only 23/9721 (0.24%) events belong to this population in this sample. SamSPECTRAL could properly identify the rare population in 27/34 (79.4%) samples from the stem cell data set.

We implemented our algorithm with R, and applied it on four different datasets. We were able to identify some types of biologically interesting populations that were previously known to be hard to distinguish, including:

1. *Overlapping populations *(Figure 4a-c).

2. *Subpopulations of a major population *(Figure 4d-f).

3. *Non-elliptical shaped populations *(Figures 5 and Figure 6a-c).

4. *Low density populations close to dense ones *(Figures 6d-f and Figure 7).

5. *Rare populations comprising less than 2% of all data points *(Figure 8).

Here, we demonstrate the capabilities of SamSPECTRAL in identifying biological populations in these cases and compare our results with two state of the art methods for clustering flow cytometry data, flowMerge (version 0.4.1) and FLAME (version 3), respectively obtained through BioConductor and GenePattern.

#### Overlapping Populations

Traditionally, identifying cell populations in flow cytometry data is accomplished by visualizing the multidimensional data as a series of bivariate plots, and separating interesting sections manually, in a process termed gating. Gating becomes challenging for high dimensional data since when the data is mapped to two dimensions, some clusters may overlap, resulting in the mixing of different populations. Consequently, even a trained operator cannot identify overlapping populations properly in all cases. However, our algorithm prevents this undesired error by considering all data dimensions together (Figure 4a-c). Model based multidimensional techniques also perform generally well in this regard.

#### Subpopulations of a Population

Figure 4d-f shows a major blood population (granulocytes) formed from two distinct subpopulations as verified by expert manual analysis. SamSPECTRAL could clearly distinguish between two subpopulations. flowMerge merged these two populations into one, while FLAME split both subpopulations.

#### Non-elliptical Shaped Populations

While most model based techniques have *a priori *assumptions on the shape of populations that resulted in mixing or splitting populations, our method worked relatively well on the samples with arbitrary shape populations. In Figure 5, the PI positive population (blue diagonal one) was clearly identified despite its non-elliptical shape. flowMerge could also distinguish this population, but it incorrectly split the PI negative population into two parts. FLAME did not correctly distinguish the two populations. Figure 6a-c shows the output of the three algorithms on a four dimensional sample from GvHD dataset. While the red population has a complex shape, it could be identified with high accuracy by SamSPECTRAL. While FLAME produced a satisfactory result, flowMerge mixed this population with the one below it.

#### Low Density Populations Close to Dense Populations

Figure 6d-f shows a sample from GvHD dataset containing a relatively low density and a high density population close together. SamSPECTRAL clearly distinguished the red population in the centre of the plot from the yellow dense population to its left. Moreover, it did not mix the red population with the other low density population to its right. FlowMerge also clustered this sample relatively well, requiring five times more processing time. The performance of FLAME was not satisfactory for this sample due to mixing the desired population with the other low density ones.

Figure 7 depicts a sample from the stem cell dataset containing a relatively low density population shown in blue. In each row, three 2-dimensional plots of the 3-dimensional data sample are presented. SamSPECTRAL could distinguish the blue population although it was surrounded by three relatively denser populations (the yellow, green and red ones). FlowMerge mixed this population with the yellow one, while FLAME mixed it with the red one.

#### Rare Populations

Identifying rare populations has many significant applications in flow cytometry experiments including distinguishing cancer stem cells, hematopoietic stem cell transplantation, detection of fetal cells in maternal blood, detection of leukocytes in leukocyte-depleted platelet products, detection of injected cells for biotherapy and malaria diagnosis [44].

Figure 8 shows a typical sample from the stem cell data set that contains a rare population in red. This population is positive for all the three markers and in each sample, it comprises between 0.1% to 2% of total cells. We performed an experiment on 34 samples from the stem cell data set and compared the performance of SamSPECTRAL, flowMerge and FLAME. This rare population was distinguished manually and the result of manual gating was considered as the basis for our comparison. FLAME and flowMerge could identify this population only in 11 (32%) and 9 (26%) of samples, respectively.

SamSPECTRAL could distinguish this population in 27 (79%) samples including all the ones that were identified by FLAME and flowMerge. In the 7 (21%) samples that SamSPECTRAL failed, the rare population of interest contained less than 0.15% of all data points.

To measure the accuracy of SamSPECTRAL, we define sensitivity and specificity as follows. For each sample, we call a cell positive if it belongs to the rare population of interest, and it is negative otherwise. Sensitivity is defined to be the number of truly identified rare cells divided by the total number of rare cells. Accordingly, specificity is the number of cells identified as negative divided by the total number of truly negative cell. The 27 (79%) cases where SamSPECTRAL correctly identified the rare population, had a 0.83 mean sensitivity with a 0.26 standard deviation. The median sensitivity was .99. Specificity was 1 except for one sample. If we consider the samples with a rare population bigger than 0.2% of the total data, we obtained median = 1, mean = 0.93 and standard deviation of 0.15 for sensitivity. A detailed report of the results of this experiment is provided as a table in additional file 1.

#### SamSPECTRAL with MCL

Figure 7j-l depicts the output of MCL on a sample from stem cells dataset. We ran MCL on the sampled data obtained by our faithful sampling algorithm and then the post-processing step was applied to the resulting clusters. This experiment showed there was no significant difference for SamSPECTRAL in clustering either through computing eigenvectors (Figure 7a-c) or by MCL (Figure 7j-l)^2^.

## Discussion

Although spectral clustering algorithm is a powerful technique, it can not be directly applied to large datasets as it is computationally expensive both in time and memory. In this study, we developed a sampling method and combined it with spectral clustering by modifying the similarity matrix based on potential theory. As a result, for the first time, analysing flow cytometry data using spectral methods becomes possible and practical. We applied SamSPECTRAL to four different flow cytometry datasets to demonstrate its applicability on a broad spectrum of flow cytometry data, and compared its performance to two state of the art model-based clustering methods optimized for flow cytometry data.

Detecting rare populations is a challenging problem and in spite of its significant applications in medical and biological research, little progress has been achieved in automatic identification of such populations. Our data reduction scheme is delicate enough not to miss rare populations comprising between 0.2% to 2% of the total data. SamSPECTRAL can identify populations of relative size in this range with acceptable accuracy.

Since our method, SamSPECTRAL, is a multidimensional clustering approach, it can identify overlapping populations that are generally hard to identify by manual gating that uses sequential two dimensional visualizations of the data. SamSPECTRAL is the first method that has demonstrated the ability to correctly identify subpopulations of major flow cytometry cell populations.

An important challenge in analysing flow cytometry data is in clustering data files that contain populations that significantly differ in density. Model-based techniques can produce errors in identifying a low density population close to denser populations because they typically make assumptions on the density of clusters [23]. Our experiments demonstrated that SamSPECTRAL can properly tackle this problem. Besides the practical observations, this capability is justified by the following observation. Spectral methodology clusters the graph such that the normal cut is "almost" optimum [41]. Now, assume that it can distinguish between two clusters when their densities are comparable. Then, if the size of the smaller cluster is reduced without change in its shape or distribution, the normal cut between them remains similar because the number of vertices and edges reduces almost proportionally to each other. Therefore, the clusters remain distinguishable. This explains why the overall performance of SamSPECTRAL is independent of cluster densities as long as their shapes are preserved.

Since parametric methods such as FLAME and flowMerge make *a priori *assumptions on the distribution or shape of the clusters [23], they may fail in identifying populations with arbitrary shapes. Although flowMerge attempts to solve this issue by finding more clusters than needed and then merging them together, it still does not produce satisfactory results when the shape of the cluster is complex. SamSPECTRAL has the capability of identifying arbitrary shape clusters since it is a non-parametric approach that makes no assumptions on the shape and distribution of clusters, and clusters data based only on similarity between data points. Compared to other non-parametric methods, our algorithm has the advantages of automatically identifying the number of clusters and having low sensitivity to the predefined thresholds. Therefore, users can adjust the parameters only once by running SamSPECTRAL on one or two random samples from a flow cytometry data set. Then, the algorithm can be run on the rest of data set without changing the parameters.

Not only does our sampling scheme increase the speed of spectral clustering without losing important biological information, but the resulting algorithm is faster than other methods considered in this study. More precisely, the running time of SamSPECTRAL is *O*(*dmn*) + *O*(*m*^3^) where *O*(*dmn*) is the running time for building *m *communities from *n *points in *d *dimension and *O*(*m*^3^) is the running time for computing the eigenspace. After this step, the k-means clustering runs very fast in time O(k m t) to find *k *clusters using eigenvectors by *t *iterations. In comparison, the time complexity of the original MCL method is *O*(*nr*^2^) with no guarantee on upper bound for number of iterations *r*, other than *n*. Practically, for our model of flow cytometry data where all pairs of data points are connected, we could not run MCL before applying our modification to it. Moreover, SamSPECTRAL running time is significantly less than model-based techniques. The running time of flowMerge is *O*(*d*^2^*k*^2^*nt*) and FLAME runs in time *O*(*d*^4^*klnt*) where *l *is the number of times it runs to find the optimal number of clusters. In practice, we can keep *m *as small as 1500-3000 without loosing important biological information, and consequently SamSPECTRAL ran at least 5-10 times faster than flowMerge and FLAME on the studied datasets. Furthermore, the time efficiency of our algorithm is more noticeable for higher dimensional data such as the one provided as additional file 2. This sample contains 100,000 events in 23 dimensions and SamSPECTRAL can analyze it in less than 25 minutes by a 2.7 GHz processor.

## Conclusions

Faithful sampling is based on potential theory. It reduces the size of input for spectral clustering algorithms and consequently they can now be efficiently applied on flow cytometry data in spite of its large size. Practically, our approach demonstrated significant advantages in proper identification of populations with non-elliptical shapes, low density populations close to dense ones, minor subpopulations of a major population, rare populations, and overlapping populations. No state of the art method can solve the challenges in identifying populations with the above properties simultaneously. Moreover, applying SamSPECTRAL to other biological data such as microarrays and protein databases may result in significant improvements in gene expression and protein classification.

Besides, our faithful sampling algorithm can have interesting applications by itself. For instance, it can be used appropriately to reduce the size of input for other clustering algorithms that are based on spectral graph theory such as Markov Clustering Algorithm (MCL), electrical circuit based clustering, and agent based graph clustering [45]. We have shown the extendibility of our approach in this sense by substituting classic spectral clustering with MCL, a method that has many applications in bioinformatics.

Other directions for future work include applying other schemes for estimating similarities between communities, combining clusters based on other combinatorial algorithms or biological criteria, and repeating the algorithm several times to obtain a more stable outcome.

## Methods

To run flowMerge and FLAME optimally, we used several settings for their parameters, finally selecting those that gave us the best results. For SamSPECTRAL algorithm, we set *m *= 3000 to keep the running time bellow 1 minute by a 2.7 GHz processor and the obtained results remained satisfactory for all samples we analyzed. The separation factor and scaling parameter (*σ*) are two main parameters that needed to be adjusted. Decreasing *σ *and increasing the separation factor will result in identifying more populations. In particular, if *σ *is decreased, then according to the heat kernel formula, the weights on the edges of the graph will decrease exponentially. Therefore, the graph will be sparser and tends to obtain more partitions. In consequence, the algorithm identifies more spectral clusters. This phenomenon can be useful in identifying rare populations. On the other hand, if separation factor is too high, a single population may be split into parts. In our experiments, we applied SamSPECTRAL on one or two random data samples of a data set and tried different values. Then, the selected parameters were fixed and used to apply SamSPECTRAL on the rest of data samples. The parameters values for the data sets presented in this paper are provided in additional file 3, 4, 5, and 6. The reader is referred to the SamSPECTRAL **Bioconductor **package vignette for more explanation on how to adjust parameters for a given data set^3^.

### Datasets

We tested our algorithm on four different flow cytometry datasets as explained briefly here. The GvHD dataset is available in flowCore package through BioConductor, and the rest are available upon request.

#### Stem Cells

To investigate heterogeneity in the differentiation behaviour of hematopoietic stem cells, a subpopulation of adult mouse bone marrow was isolated and then each single stem cell was transplanted into one of 352 recipients [46]. 16 blood samples were taken from the recipients in biweekly intervals and were studied in a cytometer. The investigation contained hundreds of data files that needed to be analyzed to count the frequency of each subtype of white cells they contained.

#### Telomere

In all vertebrates, telomeres consist of tandem DNA repeats of the sequence d(TTAGGG) and associated proteins. Telomere length is known to be crucial elements in ageing and various diseases including cancer and it can be estimated by flow cytometry [47]. Since telomere length is different for various cell populations, these need to be distinguished before calculating telomere length.

#### GvHD

Acute graft versus host disease (GvHD) is a common outcome after bone marrow transplantation. It is difficult to diagnose in its early stages in order to provide timely treatment. To investigate how flow cytometry can help predict the development of GvHD, and to study its advantages over microarrays, peripheral blood samples from 31 patients undergoing allogeneic blood and marrow transplant were analyzed [48]. The samples were taken at progressive time points post-transplant and were stained with four appropriate lymphocyte phenotypic and activation markers defining 121 different populations using six markers.

#### Viability

Propidium iodide (PI) is a widely used marker for determining viability of mammalian cells [49] because it has the capability of passing through only damaged cell membranes. However, depending on the complexity of the data, identifying dead cells automatically might still be difficult even if this marker is used. We tested the capability of our algorithm in identifying dead cells using PI marker on a dataset from the Terry Fox Laboratory.

## Authors' contributions

The authors wish it to be known that, in their opinion, the first two authors, HZ and PS, should be regarded joint first authors. HZ designed and implemented the faithful sampling algorithm, and the method for computing similarity between communities. PS developed the idea of non-uniform sampling for spectral clustering and experimentally verified the stability of the algorithm. HZ and PS jointly worked on the method for estimating the number of clusters, and post-processing steps. HZ and PS performed the experiments. HZ, PS and RB wrote the paper. RB provided data and computing facilities. AG studied the convergence of faithful sampling. AG and RB supervised the project. All authors read, edited and approved the final manuscript.

## Appendix 1

In the results section, we explained that the resolution of the sample points (Figure 2) is high enough such that by repeating the randomized faithful sampling procedure, the outcome of SamSPECTRAL does not vary significantly. The following experiment is performed to confirm this observation quantitatively. In this experiment we used F-measure, which is known to be appropriate for comparing clustering results of flow cytometry data [50]. F-measure varies in range 0-1 and reaches its best value at 1 when the two clustering results are identical. We ran SamSPECTRAL on a sample from the stem cell data set 20 times and compared the final results. The F-measure values obtained by pairwise comparison between the final results had mean = 0.98, median = 0.98 and standard deviation 0.0097.

## Appendix 2

**Figure 9:**
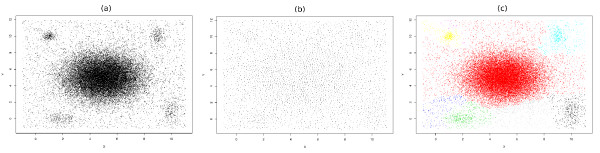
**Performance of SamSPECTRAL on synthetic data**. (a) This synthetic two dimensional data consists of a normal distribution with 30,000 points, four normal distribution each with 300 points and a uniform background noise with 4000 points. (b) Around 3000 sample points are picked up by faithful sampling. These are distributed almost uniformly in the space, therefore, almost all information about density will be lost if one considers only the samples points. (c) The final outcome of SamSPECTRAL confirms that the information about density could be retrieved by properly assigning weights to the edges of the graph. The high density cluster is shown in red and the surrounding sparser clusters are shown in yellow, light blue, green and black.

We performed the following experiment to show the effect of edge weights on performance of spectral clustering. As shown in Figure 9, we produced synthetic data containing one normal distribution with relatively high density surrounded by four relatively small clusters with lower densities. The number of points in each small cluster is less than 0.01% of the whole data and noise is added to the data space uniformly (Figure 9a). For the central dense distribution, we set *σ_xx _*= *σ_yy _*= 2,

*σ_xy _*= *σ_yx _*= 0 and the surrounding clusters are normal distributions with

$\sigma_{xx}^{1}=0.08,\;\sigma_{yy}^{1}=0.30,\;\sigma_{xy}^{1}=\sigma_{yx}^{1}=0$,

$\sigma_{xx}^{2}=0.07,\;\sigma_{yy}^{2}=0.08,\;\sigma_{xy}^{2}=\sigma_{yx}^{2}=0$,

$\sigma_{xx}^{3}=0.50,\;\sigma_{yy}^{3}=0.10,\;\sigma_{xy}^{3}=\sigma_{yx}^{3}=0$,

$\sigma_{xx}^{4}=0.10,\;\sigma_{yy}^{4}=0.70,\;\sigma_{xy}^{4}=\sigma_{yx}^{4}=0$.

The R code to produce this synthetic data and run SamSPECTRAL on it is provided in additional file 7.

After faithful sampling is done (Figure 9b), the sample points are distributed almost uniformly, and the information about the local density of original data is lost. However, faithful sampling provides us with more information than only the sample points. It will also return the members of each community and our data reduction scheme uses this information to assign weight to the edges. According to formulas 3 and 4, the more populated and closer two communities are, the higher the weight between them will be (Figure 1c). According to Figure 9c, this strategy is successful in retrieving information about local density as all the five clusters are distinguished properly by SamSPECTRAL.

## Appendix 3

**Figure 10:**
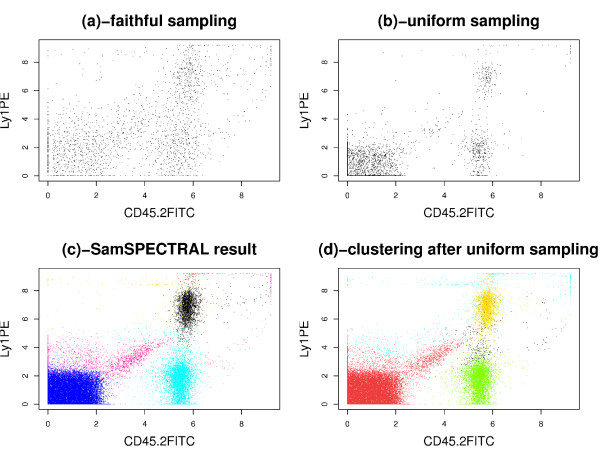
**Comparing Uniform sampling with faithful sampling**. Directly applying classical spectral clustering is not efficient on this sample of the stem cell dataset which contains 48000 cytometry events in 3 dimensions. (a) Although only 2115 data points were selected by faithful sampling, each population has a considerable number of representatives in the selected points. (b) 3000 points were selected by uniform sampling. The low density population in the middle of the plot consists of only 55 sample points resulting in mixing this population with a high density one incorrectly (d). (c) The result of SamSPECTRAL on the original data is satisfactory because the low density red population and other high density populations are identified properly.

We observed that some low density populations disappeared entirely when simple uniform sampling was employed. To investigate the effect of this phenomenon on the final clustering results, we performed an experiment on a sample of the stem cell dataset that contained 48,000 events in 3 dimensions. First, 3,000 data points were selected uniformly at random. Then, we assigned a label to each of these selected points by applying classical spectral clustering on them. Finally, for each original data point, the label of the closest selected point was considered as its cluster label. Figures 10d and 10c show the results of this approach and SamSPECTRAL, accordingly. The red population that was distinguished by SamSPECTRAL correctly in Figure 10c consists of only 4% of the data. This population could not be distinguished properly by any setting of the parameters after uniform sampling (Figure 10d).

## Footnotes

^1 ^Cheeger inequality is an example of such a relation [41].

^2 ^The CD45+ cells that are considered as outliers by MCL are not plotted in Figure 7j-l.

^3 ^The vignette is located at: http://bioconductor.org/packages/devel/bioc/vignettes/SamSPECTRAL/inst/doc/Clustering_by_SamSPECTRAL.pdf

## Supplementary Material

Additional file 1 **Report on identification of rare population**. The table contains the full detailed report on our comparative experiment for identifying rare populations. Click here for file

Additional file 2 **High dimensional flow cytometry data**. This data file contains a matrix with 100,000 rows and 23 columns that represents a flow cytometry sample with 100,000 events. It can be directly loaded in R and analyzed by SamSPECTRAL. It takes less than 12 minutes to perform faithful sampling on this 23 dimensional data. Click here for file

Additional file 3 **Parameters for GvHD data set**. These values are appropriate for running SamSPECTRAL on GvHD data set. Click here for file

Additional file 4 **Parameters for stem cell data set**. These values are appropriate for running SamSPECTRAL on stem cell data set. Click here for file

Additional file 5 **Parameters for telomere data set**. These values are appropriate for running SamSPECTRAL on telomere data set. Click here for file

Additional file 6 **Parameters for viability data set**. These values are appropriate for running SamSPECTRAL on viability data set. Click here for file

Additional file 7 **Simulation with synthetic data**. This R source code produces synthetic data with 5 clusters shown in Figure 5. The resulting data is passed to SamSPECTRAL to be clustered. Click here for file
