# Supplementary figures and images for: Data reduction for spectral clustering to analyze high throughput flow cytometry data
Source: BMC Bioinformatics. 2010 Jul 28;11:403. doi: 10.1186/1471-2105-11-403 (PMC2923634; doi:10.1186/1471-2105-11-403)

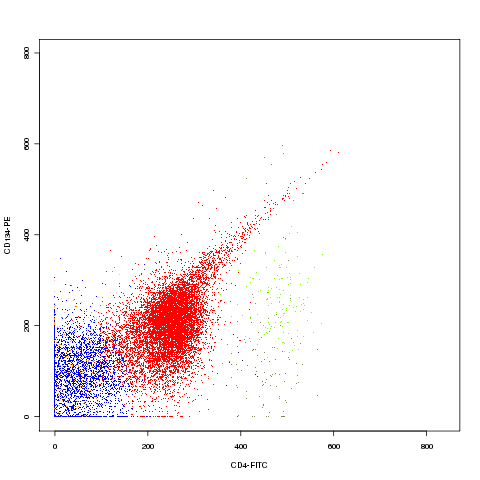

Supplement: Additional file 3 — Parameters for GvHD data set. These values are appropriate for running SamSPECTRAL on GvHD data set. [file 1471-2105-11-403-S3.zip › GvHD/GvHDA/sigma_0.00125-34.bmp]

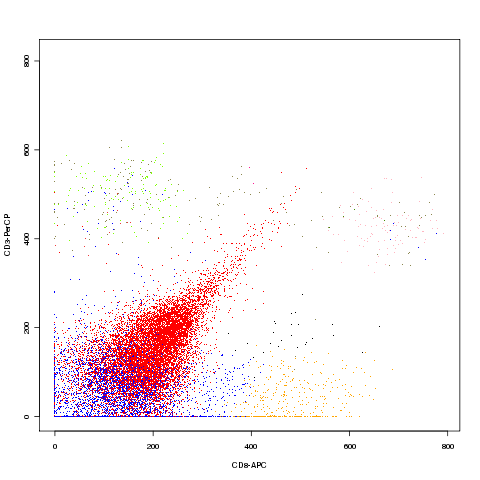

Supplement: Additional file 3 — Parameters for GvHD data set. These values are appropriate for running SamSPECTRAL on GvHD data set. [file 1471-2105-11-403-S3.zip › GvHD/GvHDA/sigma_0.00125-12.bmp]

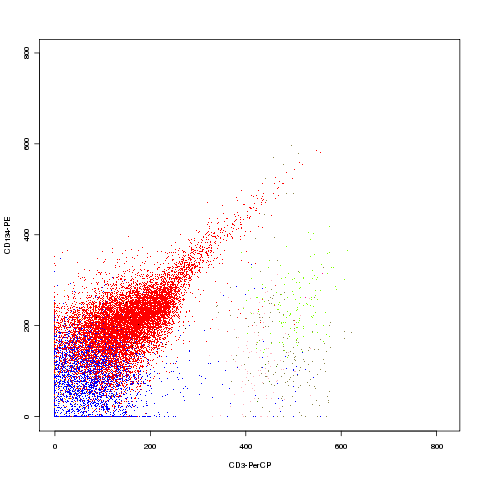

Supplement: Additional file 3 — Parameters for GvHD data set. These values are appropriate for running SamSPECTRAL on GvHD data set. [file 1471-2105-11-403-S3.zip › GvHD/GvHDA/sigma_0.00125-24.bmp]

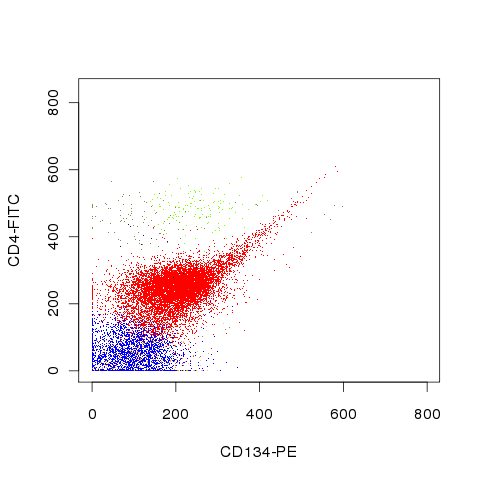

Supplement: Additional file 3 — Parameters for GvHD data set. These values are appropriate for running SamSPECTRAL on GvHD data set. [file 1471-2105-11-403-S3.zip › GvHD/GvHDA/sigma_0.00125-43.bmp]

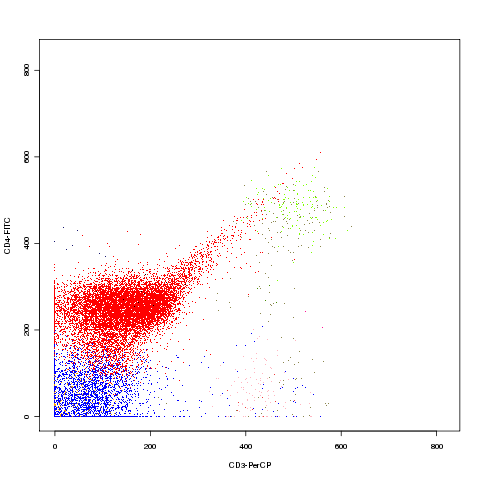

Supplement: Additional file 3 — Parameters for GvHD data set. These values are appropriate for running SamSPECTRAL on GvHD data set. [file 1471-2105-11-403-S3.zip › GvHD/GvHDA/sigma_0.00125-23.bmp]

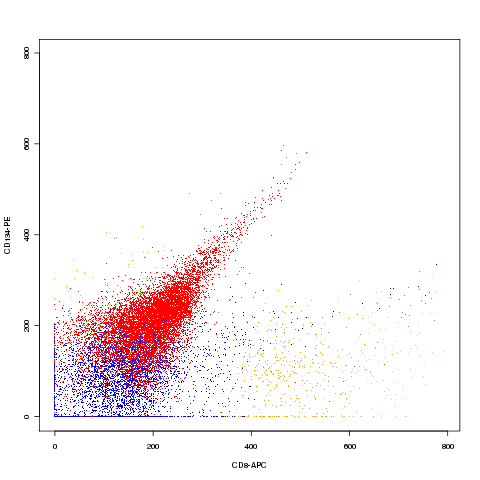

Supplement: Additional file 3 — Parameters for GvHD data set. These values are appropriate for running SamSPECTRAL on GvHD data set. [file 1471-2105-11-403-S3.zip › GvHD/GvHDA/sigma_0.00125-14.bmp]

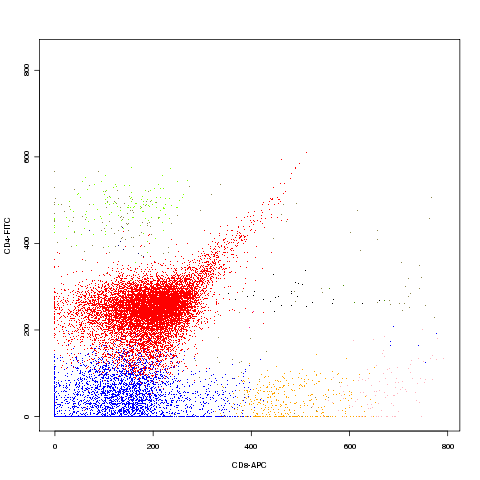

Supplement: Additional file 3 — Parameters for GvHD data set. These values are appropriate for running SamSPECTRAL on GvHD data set. [file 1471-2105-11-403-S3.zip › GvHD/GvHDA/sigma_0.00125-13.bmp]

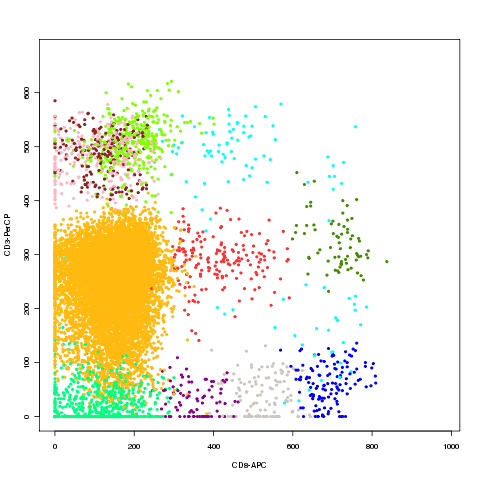

Supplement: Additional file 3 — Parameters for GvHD data set. These values are appropriate for running SamSPECTRAL on GvHD data set. [file 1471-2105-11-403-S3.zip › GvHD/GvHDB/sigma_0.00125-12.bmp]

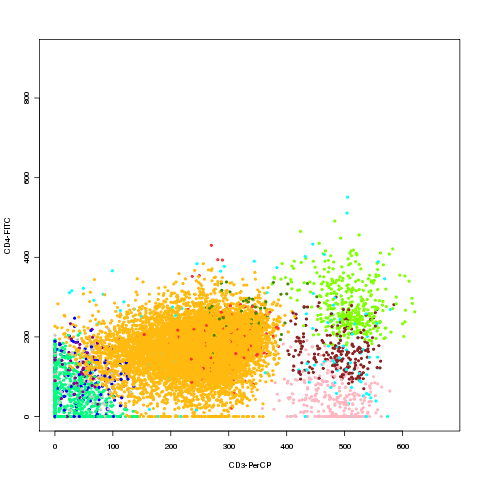

Supplement: Additional file 3 — Parameters for GvHD data set. These values are appropriate for running SamSPECTRAL on GvHD data set. [file 1471-2105-11-403-S3.zip › GvHD/GvHDB/sigma_0.00125-23.bmp]

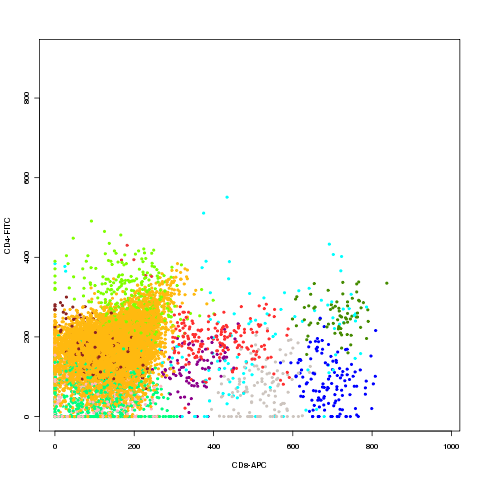

Supplement: Additional file 3 — Parameters for GvHD data set. These values are appropriate for running SamSPECTRAL on GvHD data set. [file 1471-2105-11-403-S3.zip › GvHD/GvHDB/sigma_0.00125-13.bmp]

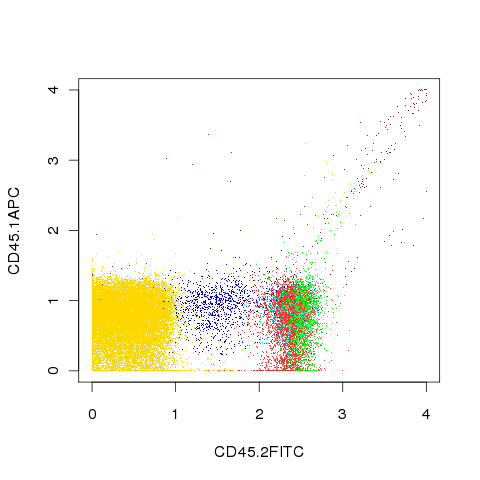

Supplement: Additional file 4 — Parameters for stem cell data set. These values are appropriate for running SamSPECTRAL on stem cell data set. [file 1471-2105-11-403-S4.zip › Stem Cells/sigma_22.2222222222222-13.bmp]

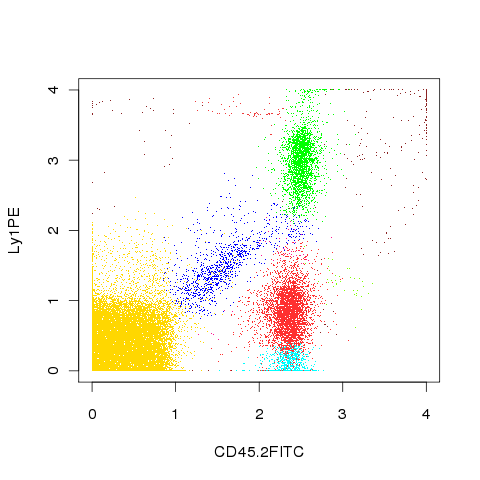

Supplement: Additional file 4 — Parameters for stem cell data set. These values are appropriate for running SamSPECTRAL on stem cell data set. [file 1471-2105-11-403-S4.zip › Stem Cells/sigma_22.2222222222222-12.bmp]

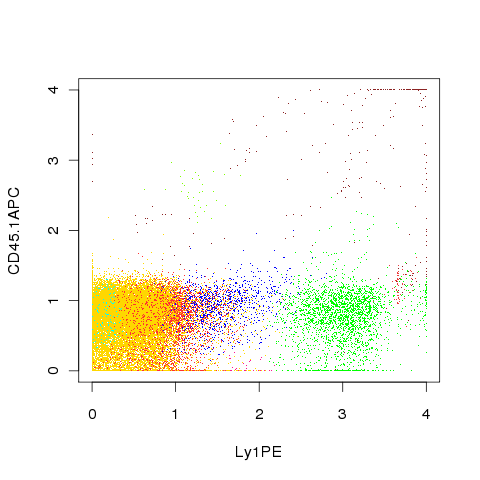

Supplement: Additional file 4 — Parameters for stem cell data set. These values are appropriate for running SamSPECTRAL on stem cell data set. [file 1471-2105-11-403-S4.zip › Stem Cells/sigma_22.2222222222222-23.bmp]

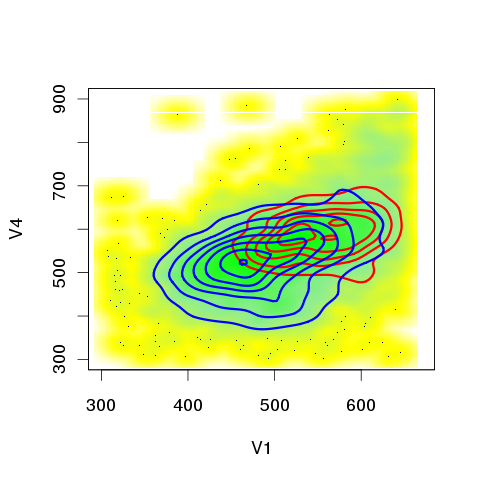

Supplement: Additional file 5 — Parameters for telomere data set. These values are appropriate for running SamSPECTRAL on telomere data set. [file 1471-2105-11-403-S5.zip › Telomere/3sigma_5e-04contours-13.bmp]

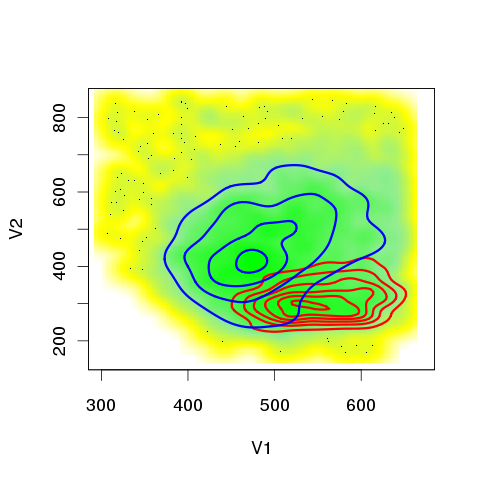

Supplement: Additional file 5 — Parameters for telomere data set. These values are appropriate for running SamSPECTRAL on telomere data set. [file 1471-2105-11-403-S5.zip › Telomere/3sigma_5e-04contours-12.bmp]

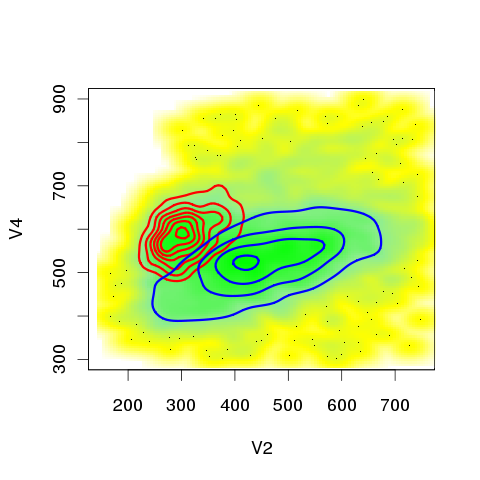

Supplement: Additional file 5 — Parameters for telomere data set. These values are appropriate for running SamSPECTRAL on telomere data set. [file 1471-2105-11-403-S5.zip › Telomere/3sigma_5e-04contours-23.bmp]

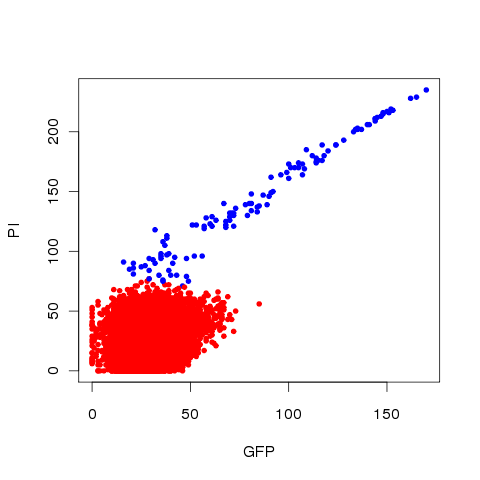

Supplement: Additional file 6 — Parameters for viability data set. These values are appropriate for running SamSPECTRAL on viability data set. [file 1471-2105-11-403-S6.zip › Viability/sigma_0.0102040816326531-12.bmp]
